# Electromyographic evidence of reduced emotion mimicry in individuals with a history of non-suicidal self-injury

**DOI:** 10.1371/journal.pone.0243860

**Published:** 2020-12-28

**Authors:** Laura Ziebell, Charles Collin, Monica Mazalu, Stéphane Rainville, Madyson Weippert, Misha Skolov

**Affiliations:** 1 Department of Psychology, University of Ottawa, Ottawa, Canada; 2 VizirLabs Consulting, Chelsea, Québec, Canada; National Institutes of Health, UNITED STATES

## Abstract

Engaging in facial emotion mimicry during social interactions encourages empathy and functions as a catalyst for interpersonal bonding. Decreased reflexive mirroring of facial expressions has been observed in individuals with different non-psychotic disorders, relative to healthy controls. Given reports of interpersonal relationship difficulties experienced by those who engage in non-suicidal self-injury (NSSI), it is of interest to explore facial emotion mimicry in individuals with a history of this behaviour (HNSSI). Among other things, this will enable us to better understand their emotion regulation and social interaction challenges. Surface facial electromyography (fEMG) was used to record the reflexive facial mimicry of 30 HNSSI and 30 controls while they passively observed a series of dynamic facial stimuli showing various facial expressions of emotion. Beginning with a neutral expression, the stimuli quickly morphed to one of 6 prototypic emotional expressions (anger, fear, surprise, disgust, happiness, or sadness). Mimicry was assessed by affixing surface electrodes to facial muscles known to exhibit a high degree of electrical activity in response to positive and negative emotions: the corrugator supercilii and the zygomaticus major. HNSSI participants, relative to controls, exhibited significantly less electrical activity in the corrugator muscle in response to viewing angry stimuli, and significantly less of an expected relaxation in muscle activity in response to viewing happy stimuli. Mirroring these results, greater endorsement of social influence as a motivator for engaging in NSSI was associated with less mimicry, and greater endorsement of emotion regulation as a motivator was associated with greater incongruent muscle response when viewing happy faces. These findings lend support to the theory that social interaction difficulties in HNSSI might be related to implicit violations of expected social rules exhibited through facial mimicry nonconformity.

## Introduction

Non-suicidal self-injury (NSSI) is broadly defined as the direct and deliberate damage to one's own body tissue in the absence of suicidal intent [1]. Common methods of NSSI include cutting, scratching, burning, biting, hitting, and skin picking [2], but do not include forms of socially sanctioned self-injury, such as tattoos, ritual scarification, or piercings [3,4]. Engaging in these behaviours typically begins during early adolescence [5] with prevalence rates up to 17% in community samples [6] and between 40–80% in clinical samples [7]. NSSI is also a robust risk factor for predicting suicidal thoughts and behaviors [8,9]. Diagnostically, NSSI currently appears as a symptom of Borderline Personality Disorder (BPD) in the Diagnostic and Statistical Manual of Mental Disorders, Fifth Edition [3]; however, the prevalence of engagement in NSSI behaviour is far higher in adolescents than are rates of BPD [6,7], suggesting that NSSI can both co-occur with BPD and exist independently of it. Consequently, it has been proposed that NSSI may be a diagnostic entity of its own, and it has been included in the third section of the DSM-5 as a condition requiring further study [3]. This highlights the need for greater research into this behaviour.

Although several motivations have been identified for engaging in NSSI (i.e. NSSI functions), many models of this behaviour postulate that it primarily serves an intrapersonal function, such as regulating emotions by reducing aversive states, like sadness or anxiety [10–12]. However, secondary motivations for this behaviour have also been identified and involve interpersonal functions, like interpersonal influence or peer-bonding, that can directly or indirectly socially reinforce NSSI [11,12]. For example, interpersonal stress, perceived criticism, and social rejection have been found to be common triggers of NSSI. Hence, a sensitivity to interpersonal stress, high emotional distress, and in some cases chronic romantic stress [13] are thought to play a role in the development and maintenance of NSSI, with self-injury being used as a means to both regulate general affective states as well as regulate emotional responses to social experiences [14].

A recent hypothesis postulates that violations of social rules may contribute to the interaction difficulties and social rejection experiences reported by many individuals who engage in NSSI [15]. For instance, interpersonal interactions are highly governed by rules that guide emotional behaviour [16]. These rules are perceived as normative by interaction partners, and even minor violations of the rules can create problems for people during interactions [17]. In particular, it has been suggested that individuals who engage in NSSI may be violating the expected social rules of interaction through nonconformity in facial mimicry [15]. This nonconformity may contribute to the increased interpersonal challenges and social rejection experiences reported by this population, which are known to trigger and maintain self-harming behaviour [18,19]

In conversation, automatically and subtly mimicking the facial expression of an interaction partner (also known as micro-expressions or automatic facial expressions), is a naturally occurring and important social phenomenon that contributes to liking and rapport building between individuals [20,21]. It is defined as the imitation of facial expressions of another person through the activation of corresponding facial muscles in the observer [22], often arising in response to an emotion. The movement of facial muscles mirroring the expression of another can be either visible (overt) or non-visible (covert), and typically occurs at an unconscious level [17]. In instances where emotion mimicry is covert, and movement of the skin is not easily perceptible, muscle activation can be measured using Electromyography (EMG). In 1982, Dimberg used EMG equipment to systematically demonstrate that individuals responded with elevated activity in the zygomaticus major muscle and corrugator supercilii muscle in response to happy and angry facial expressions respectively [23]. Dimberg’s study [23] was one of the first to suggest that individuals tend to automatically and covertly mimic the observed facial expressions of others. Although the activity of many different facial muscles can be measured, that of the zygomaticus major is primarily thought to correspond with positive emotional expressions, whereas the response of the corrugator supercilii is primarily thought to correspond with negative emotions [24–26]. The zygomaticus major is responsible for stretching the lips to create a smile, whereas the corrugator supercilii lowers the brows in response to emotions such as fear, anger, and sadness [27].

Following this early research, the mimicry-eliciting effects of other facial emotions, such as happiness, anger, sadness, fear, disgust, and surprise have been examined [28]. Results continued to confirm that happy and angry expressions produced greater EMG activity in the zygomaticus major and corrugator supercilii respectively compared to neutral expressions. The corrugator supercilii has also been found to consistently show relaxation in response to happy faces[28–31]. While evidence primarily exists for the emotion mimicry effect in corrugator supercilii and zygomaticus major muscles in response to anger and happiness [22,32], other research has found corrugator supercilii responses to fearful [33–35], sad [28,36,37], disgusted [28,38,39] and surprised faces [40,41]. However, these results are somewhat inconsistent across the literature and further investigation is continuing into the mimicry-eliciting effects of these emotional expressions. Due to the exploratory nature of this research, and to add to the above literature, our study opted to present all six basic emotions to investigate if group differences could be observed in the activity elicited in these two facial muscles. Furthermore, research has found that the intensity of facial mimicry can be modulated by stimulus type. For instance, facial mimicry is more pronounced in response to dynamic facial expressions compared to static ones [42,43].

In the past, mimicry reactions were thought to be an automatic response based on a link between perception and behaviour. Perception of a specific facial expression was thought to automatically evoke the same expression in the perceiver [44]. However, current researchers believe that emotion mimicry is more complex than this. For example, Hess and Fischer consider emotion mimicry to be a case of embodied simulation, in which a person understands the affect of another by simulating the affect in themselves [22]. This process would allow the perceiver to simulate the emotion on a motor, somatosensory, and affective level, to deduce its meaning and reward value [45,46]. This more complex view of facial mimicry is supported by data showing that it can vary across contexts, as one’s own emotional state can have an influence on emotion mimicry. For example, being in a good relationship [47] or belonging to a social group [36,48] has been found to increase facial mimicry, whereas having a negative attitude toward someone can inhibit it [48–50]. Not surprisingly, mimicking behaviours appear to be a key factor in successful interactions, as this process supports empathy and emotional understanding, as well as eliciting liking and rapport in conversational partners [20,21]. However, the imitation of positive emotions appears to be more likely to lead to liking and affiliation from others than the imitation of negative ones such as anger and disgust, whose mimicry can be viewed more negatively by others [51]. Nonetheless, the act of mimicking emotions, both positive and negative, may still help build relationships through affective empathy, which means to feel what others are feeling [52].

In contrast, suppressing the mimicry of emotions, also known as expressive suppression, has been found to have negative effects on interpersonal functioning. Expressive suppression has been linked to increased physiological responding in individuals interacting with suppressors [53], decreased liking and rapport, as well as decreased willingness to form a friendship with suppressors [53]. The mechanisms that underly the link between expressive suppression and impaired social functioning are not well articulated. However, one hypothesis is that those engaged in expressive suppression may also have impairments in perceiving the emotions of others. Emotion mimicry is thought to facilitate emotion recognition by having facial muscles function as a feedback system for an individual’s own experience of emotions [20,21,23]. Indeed, blocking facial mimicry has been shown to produce less accurate identification of happiness [45,53–55], as well as slower recognition of happy, sad and fearful emotion stimuli [56]. However, it is important to note that some studies have found no link between the degree of mimicry and emotion recognition ability [47,57]. Expressive suppression has also been identified as an emotion regulation strategy in several psychological disorders, including posttraumatic stress [58,59], eating disorders [60,61], and depressive symptomatology [62,63]. This research has shown that expressive suppression can act to paradoxically increase the experience of emotion within the suppressor [53,62].

Although substantial research exists on automatic facial expressions in individuals diagnosed with schizophrenia or depressive disorders, very little is known about individuals diagnosed with non-psychotic disorders. A systematic review conducted by Davies et al., revealed altered emotion expression across all disorders examined, with the exception of anxiety disorders [64]. In depression, decreased facial expression was mainly evident for positive affect, and in eating disorders decreased facial expression was observed in response to both positive and negative stimuli. Overall, the data included in the review pointed toward decreased facial emotion expressivity in individuals with different non-psychotic disorders [64]. Interestingly, a bias toward attenuated expression was also reported for people in remission and those at risk for mental illness, particularly schizophrenia [65,66]. In two BPD studies that explored facial emotion expression with EMG and observational coding [64]. Results from both studies reported an attenuation of positive and negative facial expressions in the BPD groups [67,68]. Two other studies examining a BPD population found no group differences for zygomaticus activity during positive emotions, but increased corrugator activity for negative emotions when pictures of disgust, anger and sadness were displayed [69,70]. However, activity of the levator labii (a muscle of the upper lip related to disgust) was noted to not differ between groups.

As demonstrated by the aforementioned literature, a crucial aspect of social interaction and emotion regulation is engaging in facial mimicry during social interactions, which encourages empathy and functions as a social catalyst between individuals. Because mirroring facial expressions of emotion is a robust effect in healthy individuals, and generally decreased facial emotion expressivity has been documented in individuals with different non-psychotic disorders, it is of interest to determine if blunted or incongruent affect in facial mimicry is observed in individuals with a history of NSSI (HNSSI). If so, altered facial expressivity may be a contributing factor to difficulties with emotion regulation and social rejection, which can trigger or perpetuate the cycle of self-harm [15].

Although no known previous EMG studies have examined emotion mimicry with a history of NSSI population, it is known that individuals with different non-psychotic disorders generally display decreased facial emotion expressivity [64]. Given reports of relationship problems frequently experienced by those who engage in NSSI [71,72], and our previous findings of deficits at the perceptual level for recognizing impoverished fear stimuli [73], it is of interest to explore whether this group displays similar attenuation in facial emotion mimicry that may contribute to social interaction problems. Additionally, this study seeks to investigate whether there is a correlation between facial emotion mimicry and motivations for engaging in NSSI. Emotion suppression has been identified as an emotion regulation strategy in several psychological disorders that paradoxically results in an increased emotional experience and negative relationship outcomes. Hence, it is hypothesized that those who more strongly endorse emotion regulation and social motivations for engaging in NSSI behaviours may also show less emotion mimicry responses. Investigating the mimicry behaviours of individuals who have engaged in NSSI will help further develop research into factors that contribute to the social problems observed in this population, an underdeveloped area of research in the literature. It will also help inform treatment strategies for clinicians working with the population, particularly because interpersonal difficulties are known triggers and maintaining factors for emotion dysregulation and self-injury.

## Materials and methods

### Participants

The study sample was composed of young adults (between 17 to 24 years of age) recruited from an undergraduate subject pool at the University of Ottawa. The University of Ottawa Ethics Review Board approved this research study. A total of 60 participants (30 HNSSI and 30 control participants) were recruited. Refer to Table 1 for demographic characteristics of the sample by group. No differences were found between HNSSI and the control groups for age, sex or ethnicity. Not surprisingly, the HNSSI group had higher rates of past comorbid depression and anxiety compared to the control group.

**Table 1:** Participant demographics.

Variable	HNSSI group (*n* = 30)	Control group (*n* = 30)	*P*
Mean Age: years	18.87 ± 1.31	18.87 ± 1.31	1
Sex: male	13% (4)	17% (5)	0.72
Ethnicity: White	57% (17)	33% (10)	0.64
Past Diagnosis:			
Depression	30% (9)	3% (1)	<0.01
GAD	47% (14)	7% (2)	<0.01
PTSD	3% (1)	0% (0)	-
OCD	3% (1)	0% (0)	-
Other	3% (1)	0% (0)	-
None	43% (13)	93.3% (28)	<0.01

#### Eligibility criteria

Pre-screening questions were used to identify a subset of the university population who had a history of NSSI but reported no history of a BPD diagnosis. In order to be included in the HNSSI group, a participant had to have engaged in intentional self-inflicted injury to the surface of his or her body at least 5 times in their lifetime, with the expectation that the injury would lead to only minor or moderate physical harm (i.e., no suicidal intent). Pre-screening questions asked, “Have you ever intentionally self-inflicted damage to the surface of your body to cause bleeding, bruising, or pain (e.g., cutting, burning, stabbing, and/or hitting), without the intent to kill yourself? Please note that this does not include ear piercing, tattooing, circumcision, or cultural healing rituals.” Potential responses included Never; Once; 2–4 times; 5 or more times. Only individuals who responded “Never” or “5 or more times” were screened in to participate as controls or HNSSI respectively. Pre-screening exclusion criteria for both HNSSI and control groups included self-injury only once or 2–4 times, as well as a self-reported diagnosis of Borderline Personality Disorder. HNSSI individuals were also excluded if their self-injury status was unclear based on their subsequent responses to the Ottawa Self Injury Inventory (OSI) or the Inventory of Statements About Self-Injury (ISAS).

All HNSSI participants reported having engaged in intentional self-inflicted injury to the surface their body at least 5 times or more within their lifetime. Nearly half of the HNSSI participants 47% (*n* = 14), reported thinking about self-injuring within the past month and 27% (*n* = 8) had actually engaged in the behavior within the month. Additionally, 70% (*n* = 21) of the HNSSI participants reported thinking about self-injuring within the past 6 months and 37% (*n* = 11) of the sample reported actually engaging in self-injury within that period.

### Measures

Socio-demographic questionnaire. This demographic questionnaire collected standard participant information such as age, gender, primary language, ethnicity, education, and current or past mental health diagnosis, health conditions, as well as medications.

#### The Ottawa self injury inventory

This questionnaire (OSI—Functions 1.1) assessed self-injurious behaviours and their functions [74]. It is a 33-item self-report measure designed to identify the psychosocial functions of NSSI. It addressed cognitive, affective, behavioural, and environmental aspects of self-injury and requires approximately 20 minutes to complete. Example questions included “why do you think you started” and “if you continue, why do you still self-injure?” and example responses include “to release unbearable tension” or “to punish myself”. Answers were provided on a 5-point scale (0 = never a reason, 2 = sometimes a reason, 4 = always a reason). This scale provided cumulative scores for the subscales of internal emotional regulation (0 to 32), external emotional regulation (0 to 12), social influence (0 to 36), and sensation seeking (0 to 12). The OSI has also been shown to be valid and reliable with excellent internal consistency scores of 0.67 to 0.87 in a university sample of young adults [75].

#### The inventory of statements about self-injury

This questionnaire (ISAS—Section II) assessed an individual’s reasons for engaging in self-injurious behaviours [76]. Section II of this questionnaire was administered as a 39-item self-report measure that assessed an individual’s reasons for engaging in self-injurious behaviours on a scale from 0 (“not relevant”) to 2 (“very relevant”). Questionnaire items begin with “when I self-harm, I am…” and example responses include “causing pain so I will stop feeling numb” or “creating a physical sign that I feel awful”. Based on previous research [77], 13 functions of NSSI were identified through this questionnaire. The scores on these 13 functions were summed (ranging from 0 to 6) to create separate factors that index interpersonal functions of NSSI (i.e., autonomy, interpersonal boundaries, interpersonal influence, peer-bonding, self-care, revenge, sensation seeking, toughness; Cronbach's alpha = 0.94) and intrapersonal functions (i.e., affect-regulation, anti-dissociation, anti-suicide, marking distress, self-punishment; Cronbach's alpha = 0.84). The interpersonal functions and intrapersonal functions factors show moderate correlation (*r* = 0.40).

### Procedure

#### Consent

Prior to the task, and after having the study described to them verbally, participants read and signed an informed consent.

#### Questionnaires

All participants completed the socio-demographic questionnaire. Two additional questionnaires, the Ottawa Self Injury Inventory (OSI) and the Inventory of Statements About Self-Injury (ISAS), were completed by the HNSSI group only. If participants screened into the HNSSI group did not report any self-injury behaviours on one or both of the OSI or ISAS questionnaires, there were removed from the study sample. Additionally, if HNSSI participants reported suicidal ideation or severe harm, by endorsing Q3, Q4, Q5, or Q6 on the OSI, a suicide protocol was implemented. Furthermore, NSSI participants were all given a list of resources in the event they wished to seek further psychological support. Questionnaires were administered prior to starting the experiment to identify any participants actively engaged in suicidal ideation, and to administer the appropriate suicidal assessment protocol to these vulnerable participants. Moreover, approximately 40 minutes elapsed between completion of the questionnaires, attaching EMG sensors, and the beginning of study administration. This lengthy time between questionnaire completion and study commencement mitigates any concerns regarding emotional arousal arising from completion of the questionnaires that might otherwise have affected participants’ reactions to the study.

#### Instructions

During the emotion mimicry task, participants’ automatic and unconscious reflections of emotional expressions were recorded via EMG measurements as they passively viewed images of emotionally expressive faces. However, to record these reactions successfully, only partial disclosure of the research intent was revealed to participants during consent and throughout testing. Participants were not explicitly informed that their emotion mimicry was being recorded with EMG electrodes. Instead, in accordance with the procedures of Achaibou, Pourtois, Schwartz, and Vuilleumier [78], and Hermans, van Wingen, Bos, Putman, and van Honk [79] participants were left blind to the exact purpose of the EMG recordings, being told only that the facial electrodes were to monitor ‘‘physiological changes” such as “skin conductance” and “ocular activities”. Participant information remained confidential and anonymous after collection and all data were labelled using a 5-digit code.

#### EMG set-up

**Fig 1:**
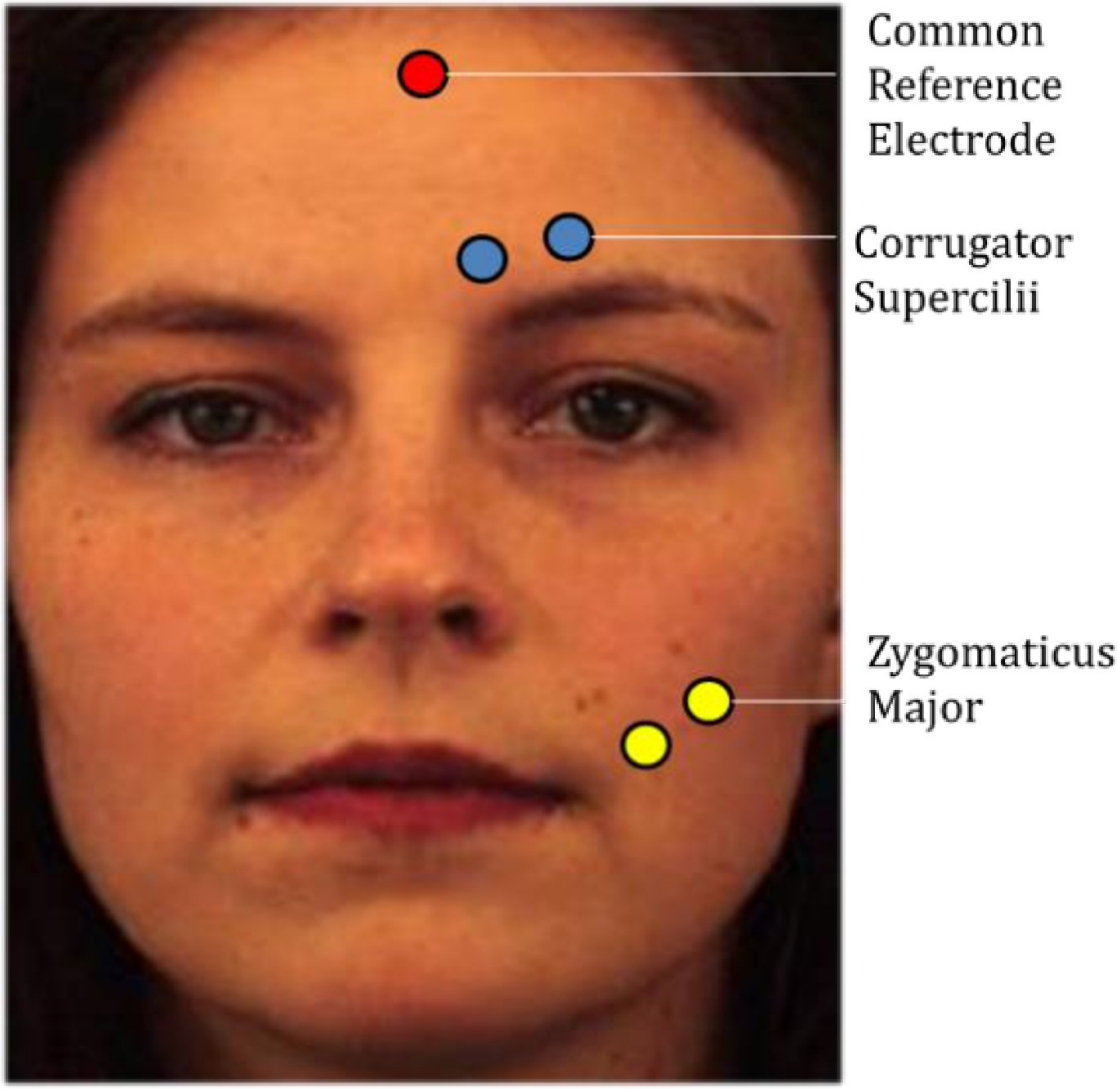
Electrode placement for measuring facial EMG. Guidelines from Fridlund and Cacioppo (1986) were used to determine correct electrode placement. This image is modified from the Karolinska Directed Emotional Faces database, with “AF06NES” displayed. Reprinted from Lundqvist, D., Flykt, A., & Öhman, A. under a CC BY license, with permission from the Karolinska Institutet original copyright (1998) [81].

The non-invasive technique of facial electromyography (fEMG) was used to evaluate and record the physiological properties of participants’ facial muscles at rest and while contracting. A Bionex™ bio-potential amplifier instrument and its electrodes (Model# 50-371102-00) were used. Facial muscle activity was measured using five Ag/AgCL miniature electrodes with a diameter of 4 mm that were externally attached to facial muscles in order to record changes in electrical potentials originating in the muscles over time and in reaction to the stimuli. Each participant’s skin was cleaned with alcohol and rubbed with an abrasive exfoliating paste to remove oils and skin residue (Lemon Prep^TM^). Pairs of electrodes were placed along the zygomaticus major and the corrugator supercilii muscle regions of the left side of the face, as recommended by Fridlund and Cacioppo [80]. A ground electrode was also placed on the upper half of the forehead (See Fig 1 for electrode placement).

Impedances were kept below 10 kΩ using a conductive EMG-gel (Signa Gel). Electromyography Analysis Software (Model 60-0103-3.1) from MindWare Technologies, LTD. (Gahanna, Ohio) was used to record the electromyography data. The EMG signal was acquired at a rate of 1000 Hz and stored on a password protected laboratory computer.

#### Emotion mimicry task

Participants were asked to passively observe a series of dynamically morphing emotional face stimuli that changed from a neutral expression to one of happiness, sadness, anger, fear, disgust or surprise. While observing the morphs, participant's facial muscle activity was recorded through electromyography. Before stimulus presentation, the experimenter instructed the participants to restrict movement as much as possible, to watch the screen continuously, and to look directly at the morph stimuli. Participants were placed in a chin rest to help reduce excessive facial and body movement. The task began with participants recording a baseline measurement of a neutral, relaxed facial expression for 20 seconds while observing a fixation cross. A series of 6 practice trials, one for each emotion expression, were then provided to ensure participants were comfortable and understood the instructions to passively observe the morphing stimuli. Following this training period, participants were presented with the experimental stimuli as their facial muscle responses were recorded. A total of 144 novel facial morphing videos were presented, each separated by a 3 second inter-trial interval fixation cross stimuli. After presenting the videos, participants were again instructed to maintain a neutral expression for 20 seconds to ensure proper electrode contact was maintained throughout the experiment. Finally, following completion of the observational task, participants were instructed to produce their maximum contraction of each muscle (corrugator supercilii and zygomaticus major) individually, for three separate trials, resulting in 6 maximum contractions, each held for 4 seconds. If a participant produced a poor contraction, an additional trial was recorded. The entire task including instructions, practice and wrap-up, took approximately 15 minutes for participants to complete.

#### Emotion mimicry stimuli

**Fig 2:**
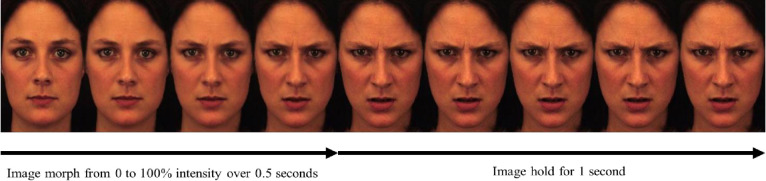
Emotion mimicry stimuli. Example stimuli intended to create a realistic simulation of dynamic emotion expressions where 150 still images were compressed into a seamless 1.5 second video progress from 0 (neutral) to 100% (prototypic expression) in 0.5 seconds then followed by a 1 second hold at full emotion intensity. These images were modified from the Karolinska Directed Emotional Faces database, with “AF01NES” and “AF01ANS” displayed. Reprinted from Lundqvist, D., Flykt, A., & Öhman, A. under a CC BY license, with permission from the Karolinska Institutet original copyright (1998) [81].

During stimulus presentation, 150 still images were compressed, using temporal interpolation, into a video of 1.5 seconds duration to produce a seamless progress from 0 (neutral) to 100% (prototypic expression) in 0.5 seconds, followed by a 1 second hold of the emotion expression at full intensity (Fig 2). This was done to create a realistic simulation of emotion expression dynamics and to evoke maximum facial mimicry in participants [42]. To create these morphing videos, a set of 24 identities (equal numbers of Caucasian male (12) and female (12) coloured photos) were selected from the Karolinska Directed Emotional Faces database [81] based on subjective image quality. The images were of amateur actors aged 20 to 30 years old with no beards, mustaches, earrings, or eyeglasses, and no visible makeup. Stimuli were pre-processed using the MATLAB image processing toolbox so that they all had equal overall lightness and color composition. A video of each of the 6 basic emotions was produced for each identity. Hence, all participants viewed 24 unique morphing videos per each of the 6 emotions (happy, sad, anger, fear, disgust, and surprise) resulting in the total of 144 facial morphing videos. These were viewed in random order.

#### Debriefing

Upon study completion, participants were given a debriefing form describing the true purpose of the EMG electrodes. Participants were then provided with the opportunity to provide complete informed consent, thus allowing for inclusion of their data in the study if they agreed, or withdrawal of their data from the study if they did not. All participants opted to have their data included. All subjects were also asked to indicate if they were aware of the true intent to record their facial muscle responses and of any (voluntary or involuntary) facial mimicry that they perceived. The HNSSI (*M* = 0.47, *SD* = 0.51) and control groups (*M* = 0.57, *SD* = 0.51) did not significantly differ in their level of awareness of the true purpose of the facial muscle recordings *t*(58) = -0.77, *p* = 0.45. Similarly, the HNSSI (*M* = 0.43, *SD* = 0.51) and control groups (*M* = 0.57, *SD* = 0.51) did not differ in their self-perception of facial muscle mimicry *t*(58) = -1.03, *p* = 0.31.

#### EMG data reduction

Off-line analyses of the EMG activity were conducted with MATLAB. The raw signal was filtered with a band-pass filter between 30 Hz to 500 Hz with a 60 Hz notch filter to account for the frequency of the connected power supply and any external electrical activity. Subsequently, the data was rectified by implementing a Hilbert rectification to conserve all signal energy and convert the signal to positive polarity [82]. A baseline correction was then applied by subtracting mean signal amplitude from the 2 s prior to stimulus presentation for each epoch. An epoch rejection algorithm was then applied to the data using 3 SD from the mean as a rejection criterion and a 50-ms shift. Visual inspection of the data was used to confirm the effectiveness of the artifact rejection process. The rejection rate was < 5% of cases for both the corrugator and zygomaticus signals. The EMG signal was smoothed using a 250ms moving average, and EMG activity over the 24 trials for each of the six emotion conditions were collapsed and averaged for each participant. This allowed us to compare EMG activity across time during the 1500ms stimulus presentation. The processed EMG data was then imported into SPSS 24 for further statistical analysis.

## Data analysis

### Power analysis

A priori power analyses were conducted using GPower 3.1.9.2 to determine the sample size required for adequate power to detect predicted differences. Several prior studies have used EMG recordings to elicit emotion mimicry reactions in various non-psychiatric populations. A meta-analysis, by Davies et al. [64] examining facial reactions to emotionally expressive face stimuli, reported Cohen’s D effect sizes based on the activation of the zygomaticus muscle in response to emotion stimuli. These Cohen’s D values varied between 0.06 and 0.78, depending on the study and population tested [83–87]. Likewise, the Cohen’s D effect sizes reported in the meta-analysis for the activation of the corrugator muscle varied widely, between 0.08 to 0.66, depending on the study [84,86,88]. Due to the variability in the effect sizes reported above, a medium effect size f = 0.25 (Cohen’s D = 0.5) was selected. Additional parameters entered into the analysis were a power of 0.8, an α of 0.05, and a mixed factorial design with a 2 (NSSI and Control, between subjects) x 6 (happy, sad, anger, fear, disgust, and surprise, within subjects) structure. The power analysis yielded an estimated minimum total sample size of 20 participants (10 control and 10 NSSI).

A second a priori power analysis was calculated using GPower 3.1.9.2 to determine the minimum sample size needed to detect a possible correlation between emotion mimicry, as measured by fEMG amplitude values, and motivations for engaging in NSSI, obtained by the OSI and ISAS questionnaires. Given that little research has been previously conducted to inform the selected effect size, a medium Cohen’s effect size for Pearson’s r (Correlation ρ H1 = 0.31) was selected with a power of 0.8 and α of 0.05 for a two-tailed correlational analysis. This calculation yielded a total sample size of 79 NSSI participants. Given that a smaller than predicted sample size was recruited due to population limitations, it is possible that the study may not have achieved sufficient power to observe the hypothesized effects for the second analysis. With our samples size of 30 participants, α of 0.05 and power of 0.8, GPower 3.1.9.2 predicted the minimum correlations size that could be detected as significant would be 0.485.

## Results

### Emotion mimicry

To analyze between-groups differences in the degree of facial EMG elicited in participants in response to observing the various emotional expression stimuli, a series of 12 planned contrasts were conducted between the HNSSI group and the control group, one for each of the 6 emotions presented and each of the 2 muscles whose activity was measured [89]. The error term for these analyses were taken from a series of 2 (Group: NSSI vs. controls) x 6 (Time bin: 0-250ms, 251-500ms, 501-750ms, 751-1000ms, 1000ms-1250ms, 1251- 1500ms) x 6 facial expression categories (happy, sad, anger, fear, disgust, surprise) mixed ANOVAs, one for each of the 2 facial muscles (refer to Tables 2 and 3).

**Table 2:** Calculation of error term from mixed factorial ANOVA for corrugator supercilii EMG activity.

Source	*df*	*MS*	*F*	η^2^_p_	*p*
Group	1	5.304^e-5^	3.400 ^e-5^	5.877 ^e-7^	0.995
Emotion	2.56	10.02	11.27	0.163	> 0.001
Emotion x Group	2.56	1.766	1.988	0.033	0.128
Error	148.48	0.888			
Time	2.282	1.94	4.205	0.068	0.013
Time x Group	2.282	0.103	0.222	0.004	0.829
Error	132.33	0.461			
Emotion x Time	7.498	1.096	5.925	0.093	> 0.001
Emotion x Time x Group	7.498	0.258	1.396	0.024	0.200
Error	434.90	0.185			

**Table 3:** Calculation of error term from mixed factorial ANOVA for zygomaticus major EMG activity.

Source	*df*	*MS*	*F*	η^2^_p_	*p*
Group	1	0.034	0.155	0.003	0.695
Emotion	3.979	0.101	0.515	0.009	0.724
Emotion x Group	3.979	0.119	0.603	0.010	0.660
Error	230.77	0.197			
Time	2.849	0.078	2.390	0.040	0.074
Time x Group	2.849	0.062	1.906	0.032	0.134
Error	165.22	0.032			
Emotion x Time	4.336	0.054	0.674	0.011	0.623
Emotion x Time x Group	4.336	0.040	0.494	0.008	0.755
Error	251.51	0.081			

In order to obtain the error term for the planned contrasts conducted between the HNSSI and the control groups for the corrugator supercilii muscle [89], a 2 (Group: NSSI or Control) × 6 (Time bin: 0-250ms, 251-500ms, 501-750ms, 751-1000ms, 1000ms-1250ms, 1251- 1500ms) x 6 (Facial expression category: happy, sad, anger, fear, disgust, surprise) mixed factorial ANOVA was conducted. Mauchly’s test indicated that the assumption of sphericity had been violated, and therefore the degrees of freedom were corrected using Greenhouse-Geisser estimates of sphericity (ε < 0.75).

The error term for the planned contrasts conducted between the HNSSI and the control groups for the zygomaticus major muscle [89], a 2 (Group: NSSI or Control) × 6 (Time bin: 0-250ms, 251-500ms, 501-750ms, 751-1000ms, 1000ms-1250ms, 1251- 1500ms) x 6 (Facial expression category: happy, sad, anger, fear, disgust, surprise) mixed factorial ANOVA was conducted. Mauchly’s test indicated that the assumption of sphericity had been violated, and therefore the degrees of freedom were corrected using Greenhouse-Geisser estimates of sphericity (ε < 0.75).

Thus, for each of the two facial muscles, six planned contrasts were performed, one for each emotion across time. Considering that only a small number of planned contrasts were conducted relative to the total number of possible comparisons, the alpha level was not adjusted and remained at α = 0.05 [89]. Effect sizes for contrasts were measured using correlation coefficient *r* [89]. The results of these contrast analyses are reported in the text.

The planned contrasts revealed attenuated EMG activity in the corrugator supercilii within the HNSSI group in response to the anger stimuli (F [1, 434.89] = 7.04, *p <* 0.008, *r =* 0.126). Likewise, the response of the corrugator supercilii was analyzed during presentation of happy facial stimuli. The typical mimicry behaviour of the corrugator supercilii is to decrease activity in response to happy faces [28]. Planned contrasts were again used to compare EMG activity of the corrugator supercilii between groups and also revealed attenuated EMG activity of the HNSSI group throughout the presentation of the happy stimuli (F [1, 434.89] = 15.84, *p <* 0.000, *r =* 0.187), as indicated by the negative polarity of the signal representing a decrease in activity, or relaxation, compared to baseline.

**Fig 3:**
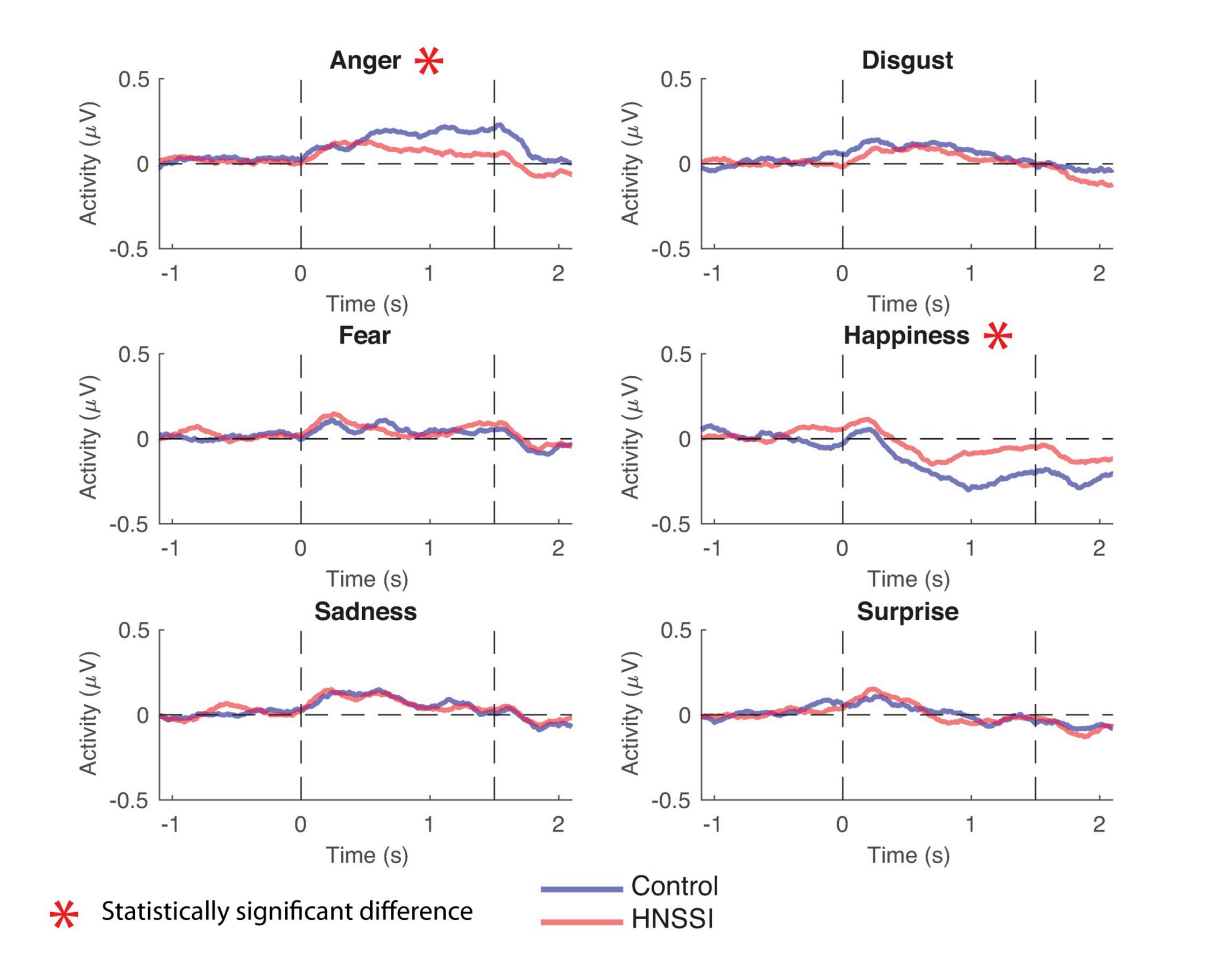
Facial EMG activity of control and HNSSI participants in the corrugator supercilii muscle. Positive values indicate increased activity compared to baseline and negative values indicate decreased activity compared to baseline, signaling muscle relaxation. The HNSSI participants showed less corrugator supercilii activity in response to viewing angry facial stimuli and less of an expected decrease in activity of the corrugator supercilii in response to viewing happy faces.

These results illustrate that the HNSSI group did not exhibit as strong a mimicry response in the corrugator supercilii muscle when observing angry and happy emotion stimuli, as compared to the control group in response to the same stimuli (Fig 3).

The EMG activity of corrugator supercilii was also analyzed in response to the presentation of the remaining four emotions (sadness, fear, surprise and disgust), but no statistically significant results were observed. Similarly, the response of the zygomaticus major was compared between the HNSSI and control groups in response to viewing all six emotions. Little mimicry was observed in this muscle in response to the stimuli presented and no statistically significant differences were observed between the HNSSI and control groups.

### Emotion mimicry and NSSI functions

Correlational analyses were conducted to determine if zygomatic and corrugator EMG activity in response to viewing facial expressions of emotion were linked to the functions reported by HNSSI participants for engaging in the behaviour. In particular, scores for the internal emotion regulation and social influence functions of the OSI questionnaire and scores for the affect regulation and peer bonding functions of the ISAS questionnaire were analyzed for their degree of correlation with participant’s overall zygomatic and corrugator EMG activity in response to viewing happy and angry facial stimuli. Correlation results for the OSI questionnaire showed that scores of emotion regulation as a motivation for engaging in NSSI were positively related to stronger contraction of the corrugator muscle in response to viewing happy facial expressions (r = 0.375, p = 0.04). Recall that the typical behaviour of the corrugator supercilii in response to viewing happy faces is to relax. Hence, this response shows greater incongruent facial mimicry than would be typically expected with greater endorsement of emotion regulation as a motivation for self-injury. In contrast, the social influence scale of the OSI was negatively correlated with zygomatic EMG muscle activity in response to viewing happy facial expressions (r = -0.382, p = 0.04). This result suggests that greater endorsement of the social influence motivation is associated with less mimicry. Likewise, peer bonding as a motivator for engaging in NSSI, as measured by the ISAS, showed a trend toward negatively correlating with EMG activity of the corrugator muscle in response to viewing angry facial expressions (r = -0.361, p = 0.05). These findings provide preliminary evidence that engaging in NSSI as a means to socially influence or bond with others may be linked with reduced mimicry of the zygomatic and corrugator facial muscles.

## Conclusion

To our knowledge, the present study is the first to investigate the facial mimicry responses of an HNSSI population in reaction to observing realistic depictions of emotional facial expressions. Several findings emerged from this study. First, attenuated EMG activity was observed in the corrugator supercilii in our HNSSI sample in response to observing angry and happy facial expressions of emotion. Second, we found evidence in support of the hypothesis that different motivations for engaging in NSSI are associated with different facial reactions to observing facial expressions of emotion. Specifically, the more strongly individuals with a history of NSSI endorsed self-injury as a means of social influence, the less mimicry they exhibited in response to happy and angry stimuli in the zygomaticus major and the corrugator supercilii, respectively. Furthermore, in HNSSI individuals, the degree to which emotion regulation was endorsed as a motivation for self-injury was found to be correlated with an incongruent mimicry response of the corrugator facial muscle in response to happy stimuli.

These findings support the hypothesis that social interaction difficulties of those who have engaged in NSSI may be related to implicit violations of expected social rules through facial mimicry nonconformity. That is, the non-verbal signs of facial emotion mimicry induced by facial stimuli are atypical in HNSSI participants compared to controls, and this divergence could play an important role in the disturbed social interactions reported by many individuals who have engaged in self-injury [90–92]. The first diverging mimicry response observed in the HNSSI group was the attenuated response in the corrugator supercilii muscle when viewing angry faces. The expected contraction of this muscle was significantly less compared to the control group. Additionally, in response to viewing happy faces, the corrugator muscle of the HNSSI group showed significantly less of an expected relaxation compared to the control group. Recall that a relaxation of the corrugator is a consistently observed response in this muscle when viewing happy faces [28–31]. The contraction and relaxation of the corrugator in response to these two emotions are typically observed in the emotion mimicry literature, yet the HNSSI group showed less of a response.

One possible explanation for the finding of attenuated facial mimicry in HNSSI individuals stems from Linehan’s (1993) theory as to the etiology of this behaviour. According to Linehan, the creator of dialectical behavior therapy for BPD, people engage in self-injurious behaviours because they do not appropriately recognize or manage negative emotions that arise from aversive mental states or external events. This inability to manage negative emotions is hypothesized to be the result of both a biological disposition and an emotionally invalidating environment, in which a child’s emotions (particularly negative emotions) were neither recognized nor validated by their parents. Consequently, children whose emotions were either ignored or even punished, likely failed to develop adaptive strategies to regulate their feelings and learned to use NSSI as a way to restore their emotional arousal to a tolerable level [93]. These children may have also learned to hide their overt facial expressions, causing the degree to which they also reflexively mimic expressions or produce micro-expressions to become suppressed later on.

Based on this theory one can posit that, because facial expressions communicate intent, individuals raised in an emotionally invalidating environment might have learned to adopt a more consistently neutral facial expression as a means to protect themselves from rejection by being less “visible” to their interaction partners, and by being perceived as less engaged in interactions. That is, since showing signs of emotion leaves a person vulnerable, particularly if the emotion displayed is not reciprocated or is dismissed by others as invalid or inappropriate, reduced facial expression mimicry may be a way for HNSSI individuals to protect themselves from signs of social rejection or scrutiny [94].

While the above explanation works well with regards to reduced mimicry of negative stimuli (e.g., angry faces), it is less clear why individuals with a history of NSSI would also mimic positive emotions less (e.g., happy faces) by relaxing the brow. Mimicking negative emotions tends to be viewed more negatively by others [51], so suppressing one’s tendency to reflect them makes sense as a measure for avoiding rejection or negative judgment. However, mimicking of positive emotions tends to be viewed positively and to increase feelings of liking and affiliation from others [20,21], so it is less clear why those with a history of NSSI would exhibit reduced mimicry in this case. One possibility is that these individuals have taken on a general strategy of not displaying any emotion, so as to hide all information about their emotional status from others. This strategy might be considered adaptive to hide one’s negative emotional states. This in turn may explain the trend in our results showing that peer bonding as a motivator for NSSI is associated with less EMG activity in the corrugator muscle in response to viewing angry facial expressions. However, in a situation where exhibiting a positive emotion may be socially expected, not doing so could easily be interpreted by others as reflecting an overall negative state. Again, our results found that greater endorsement of social influence as a motivator for engaging in NSSI was associated with less activity in the zygomatic muscle in response to viewing happy facial stimuli. In short, HNSSI individuals may have learned to hide their reflection of all emotions as a form of self-protection.

Considered through this lens, it becomes clear how such a self-reinforcing pattern of reduced spontaneous mimicry of facial expressions could contribute to ongoing social interaction difficulties frequently reported by individuals with a HNSSI [69,95]. This hypothesis might be taken to predict attenuated responses to all the emotions presented. However, our study showed only the typically observed results of mimicry effects in the corrugator supercilii muscles in response to angry and happy emotion stimuli [22]. Recall that the majority of studies have found elevated activity in the zygomaticus major and corrugator supercilii muscles in response to happy and angry facial expressions respectively [22], and many studies have found relaxation of the corrugator supercilii in response to happy faces [28–31]. Although some studies report corrugator activity to other emotions presented, we did not observe these differences. The fact that corrugator activity to emotions other than happiness and anger is only observed in some studies and not others may indicate that these effects are weak, which may in turn explain why we did not observe them. To investigate the above hypothesis further, other muscle groups that may better index the mimicry responses of disgust, sadness, fear and surprise should be examined. As such, future investigations into mimicry differences are encouraged to record activity in the frontalis for fear and surprise, the levator labii for disgust, and the mentalis for sadness. Recordings in these muscle groups may prove to be a better index for the mimicry response of these emotions and could further highlight possible group differences like those seen in the corrugator.

Another potential explanation for our results may be that some individuals with HNSSI have a greater tendency to exhibit mixed or ambiguous facial emotion expressions. Staebler et al. [70], found that a sample of participants with a diagnosis of BPD, as compared to healthy controls, tended to more often exhibit a blend of basic emotional expressions (e.g., flexing both the zygomaticus major and the corrugator supercilii) when faced with situations inducing feelings of inclusion or rejection. If this finding generalizes to the motivations for why people engage in NSSI, then it may be the case that those who more strongly endorsed emotion regulation as a motivation for NSSI in our sample were more likely to have an incongruent muscle response of contracting the corrugator muscle, as opposed to relaxing it, when viewing happy stimuli because their muscles reflected an experience of mixed emotions in response to signs of social inclusion. This hypothesis could be a suggestion for future research, as a greater number of research participants would be needed to fully investigate this theory. Also note that this explanation is not mutually exclusive to one based on incongruent mimicry. Indeed, it could well be that our results reflect some combination of attenuated and incongruous mimicry on the part of individuals with HNSSI. Either of these kinds of behaviours, or any combination of them, would be expected to lead to difficulties with social interactions.

Like all studies, several limitations of the current study need to be considered in interpreting its results. First, due to the cross-sectional design of this study, and the analysis using correlational methods, we cannot ascertain causation from the results. Second, the self-reported data collected from participants is inherently vulnerable to response bias. Since self-harm is a sensitive topic, participants may have been reluctant to report honestly about their self-injuring behaviors or motivations for engaging in the behaviour. Also, some participants may have felt stigma for endorsing interpersonal functions, such as eliciting attention from others, and thus may have under-reported interpersonal reasons as a motivation for self-injury. Third, our sample was collected from university students, which limits the generalizability of these results to other populations. Fourth, as NSSI is highly co-morbid with other disorders, it is pervasive in the literature that studies investigating NSSI include participants who have current or past co-morbid disorders. Although our sample size did not allow for examination of the potential confounding variable of comorbidity, it will be important for future studies to describe any possible influence of comorbid disorders on HNSSI emotion mimicry responses. Fifth, because this study is the first of its kind, our data need to be replicated in an independent and larger sample before they can be considered definitive.

Since a history of self-injury is an important clinical marker for increased risk of suicide, it is especially pertinent to better understanding its etiology [96]. Social dysfunctions are known to play an important role in initiating NSSI [97], and continued difficulties with social interactions often maintain the behaviour [19]. However, research into fully understanding the factors that contribute to these social interaction difficulties in NSSI is still in its infancy. Results from this study provide support for the recommendation to place greater emphasis on appropriate emotion expression and social skills training when working with this population as a complement to existing treatment approaches for NSSI in clinical practice.

## Supporting information

S1 Fig (JPG)

S2 Fig (JPG)

S3 Fig (JPG)

S1 Data Correlational analysis publication data. (XLSX)

S2 Data EMG publication data. (XLSX)
