# Supplementary figures and images for: Electromyographic evidence of reduced emotion mimicry in individuals with a history of non-suicidal self-injury
Source: PLoS One. 2020 Dec 28;15(12):e0243860. doi: 10.1371/journal.pone.0243860 (PMC7769269; doi:10.1371/journal.pone.0243860)

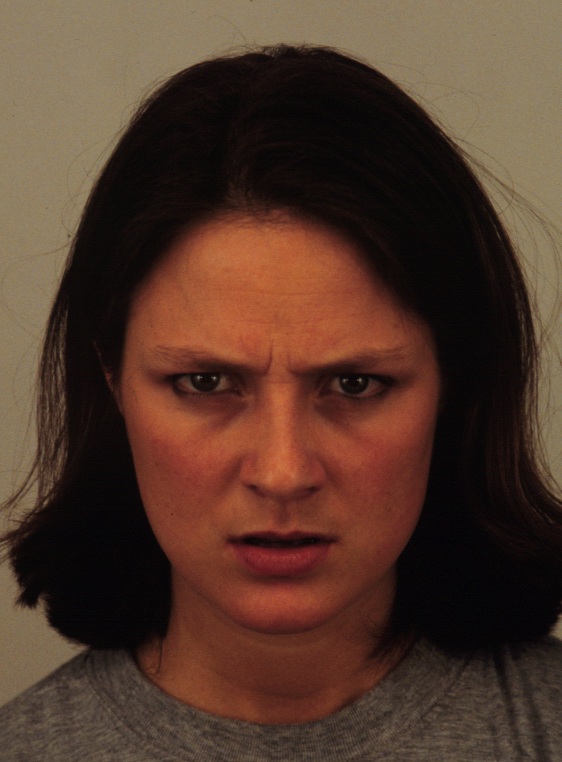

Supplement: S1 Fig — (JPG) [file pone.0243860.s001.JPG]

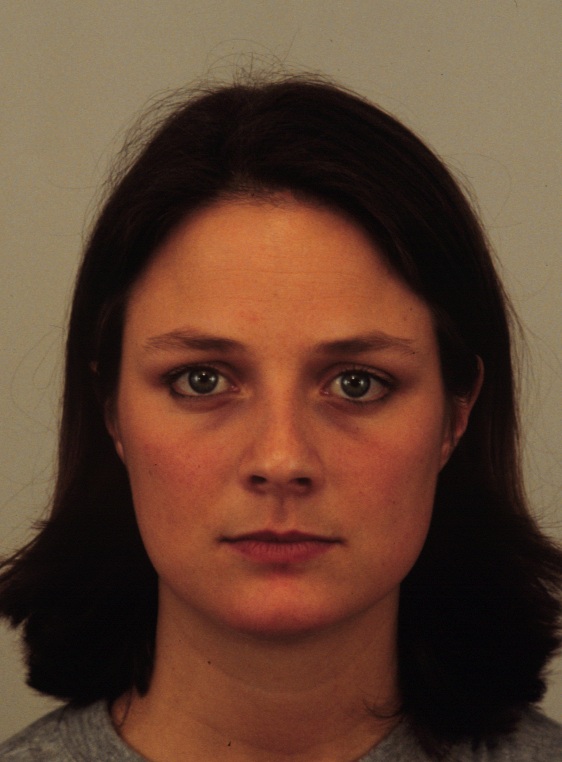

Supplement: S2 Fig — (JPG) [file pone.0243860.s002.JPG]

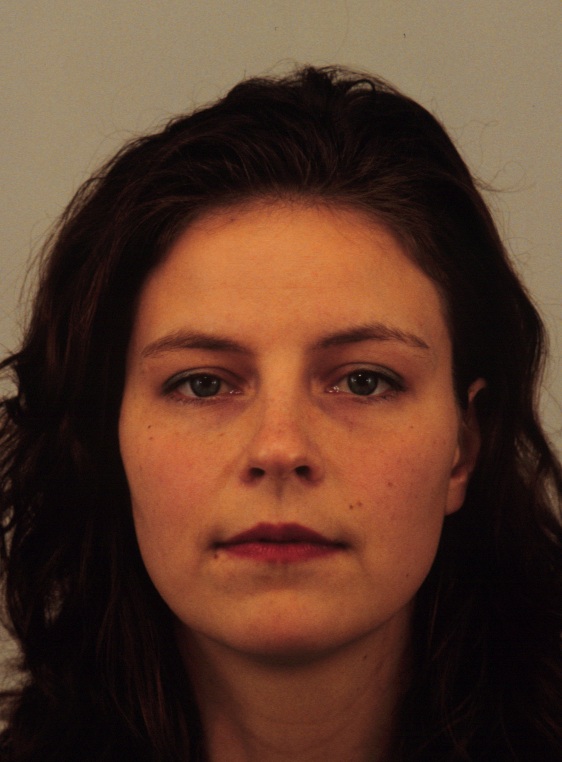

Supplement: S3 Fig — (JPG) [file pone.0243860.s003.JPG]
